# Supplementary figures and images for: Effect of ovarian stimulation on the expression of piRNA pathway proteins
Source: PLoS One. 2020 May 4;15(5):e0232629. doi: 10.1371/journal.pone.0232629 (PMC7197780; doi:10.1371/journal.pone.0232629)

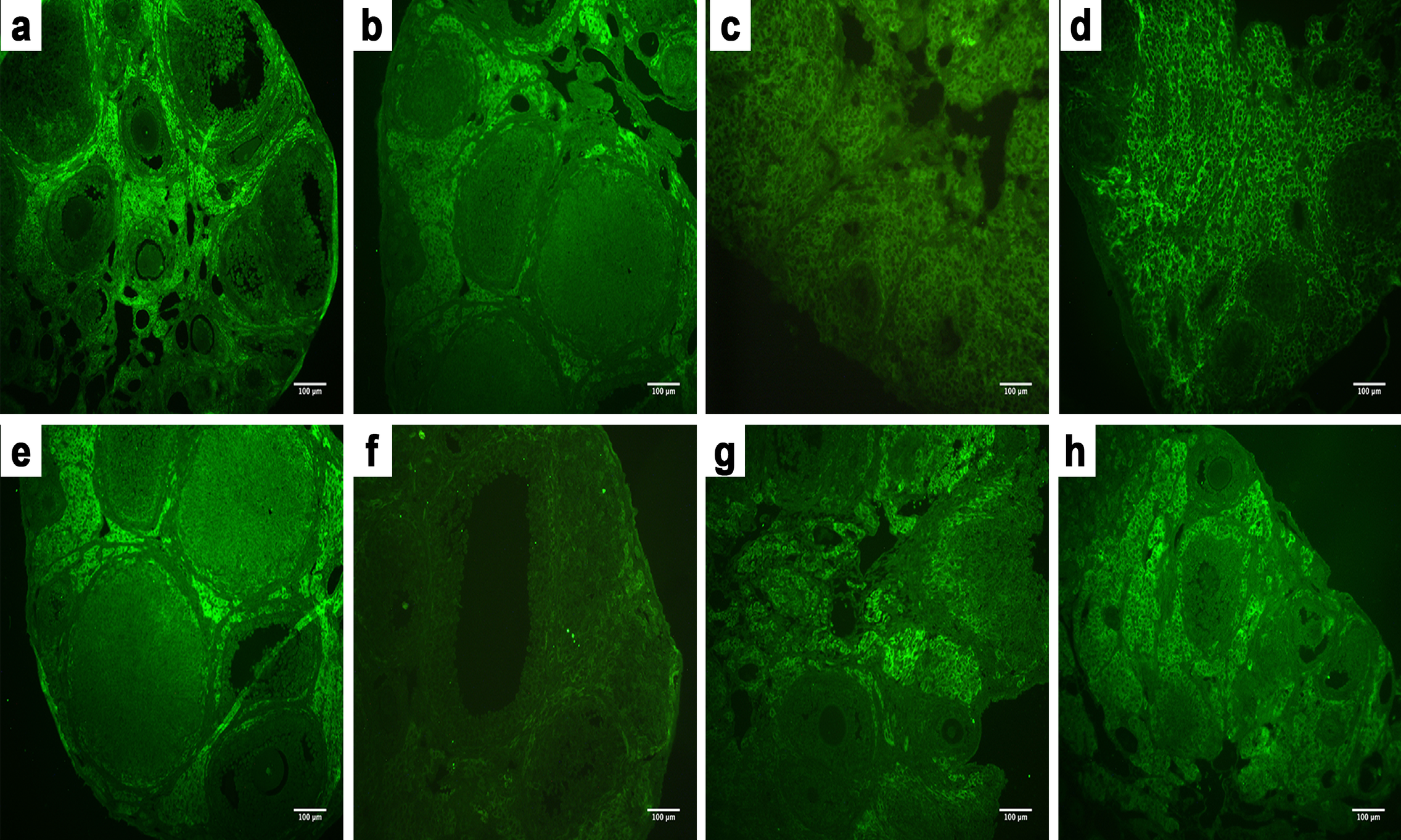

Supplement: S1 Fig — The expression and distribution of TDRD9 (stained with FITC, green) in the control (a), group 1 (b), group 2 (c), group 3 (d), group 4 (e), group 2R (f), group 3R (g), and group 4R (h) were evaluated by immunofluorescence staining (200X magnification). Green = FITC. (TIF) [file pone.0232629.s001.tif]

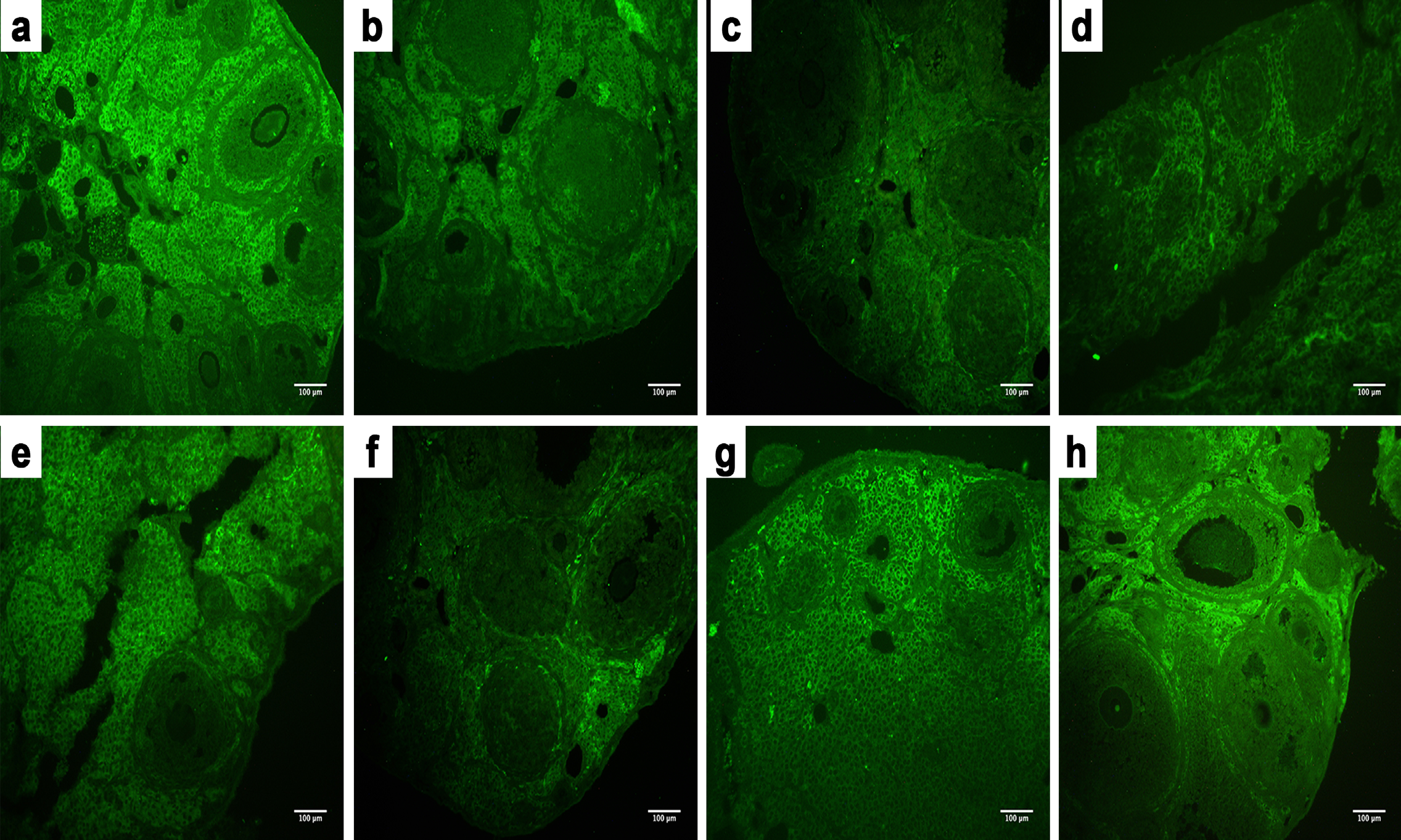

Supplement: S2 Fig — The expression and distribution of TDRD1 (stained with FITC, green) in the control (a), group 1 (b), group 2 (c), group 3 (d), group 4 (e), group 2R (f), group 3R (g), and group 4R (h) were evaluated by immunofluorescence staining (200X magnification). Green = FITC. (TIF) [file pone.0232629.s002.tif]

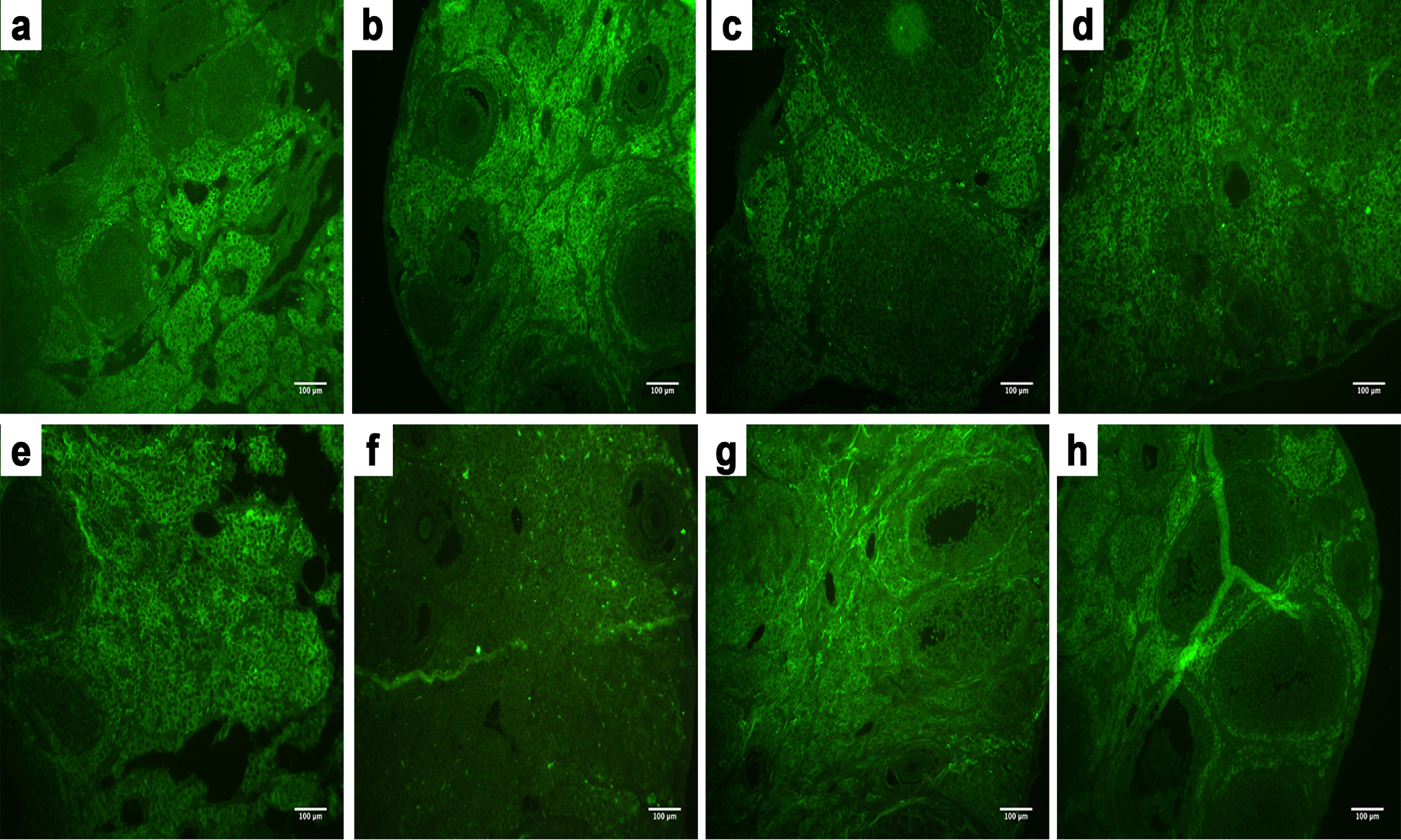

Supplement: S3 Fig — The expression and distribution of MAEL (stained with FITC, green) in the control (a), group 1 (b), group 2 (c), group 3 (d), group 4 (e), group 2R (f), group 3R (g), and group 4R (h) were evaluated by immunofluorescence staining (200X magnification). Green = FITC. (TIF) [file pone.0232629.s003.tif]

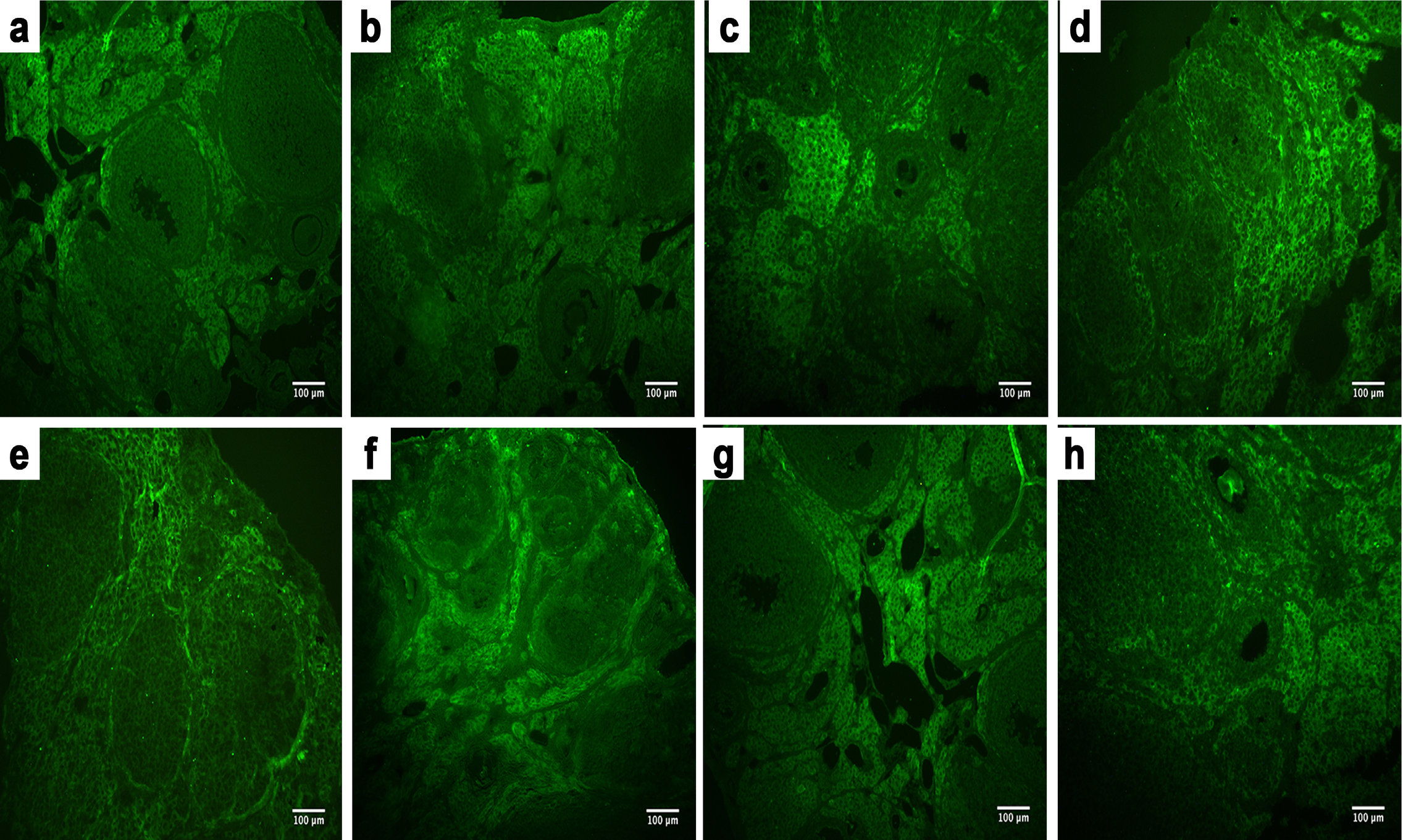

Supplement: S4 Fig — The expression and distribution of MITOPLD (stained with FITC, green) in the control (a), group 1 (b), group 2 (c), group 3 (d), group 4 (e), group 2R (f), group 3R (g), and group 4R (h) were evaluated by immunofluorescence staining (200X magnification). Green = FITC. (TIF) [file pone.0232629.s004.tif]

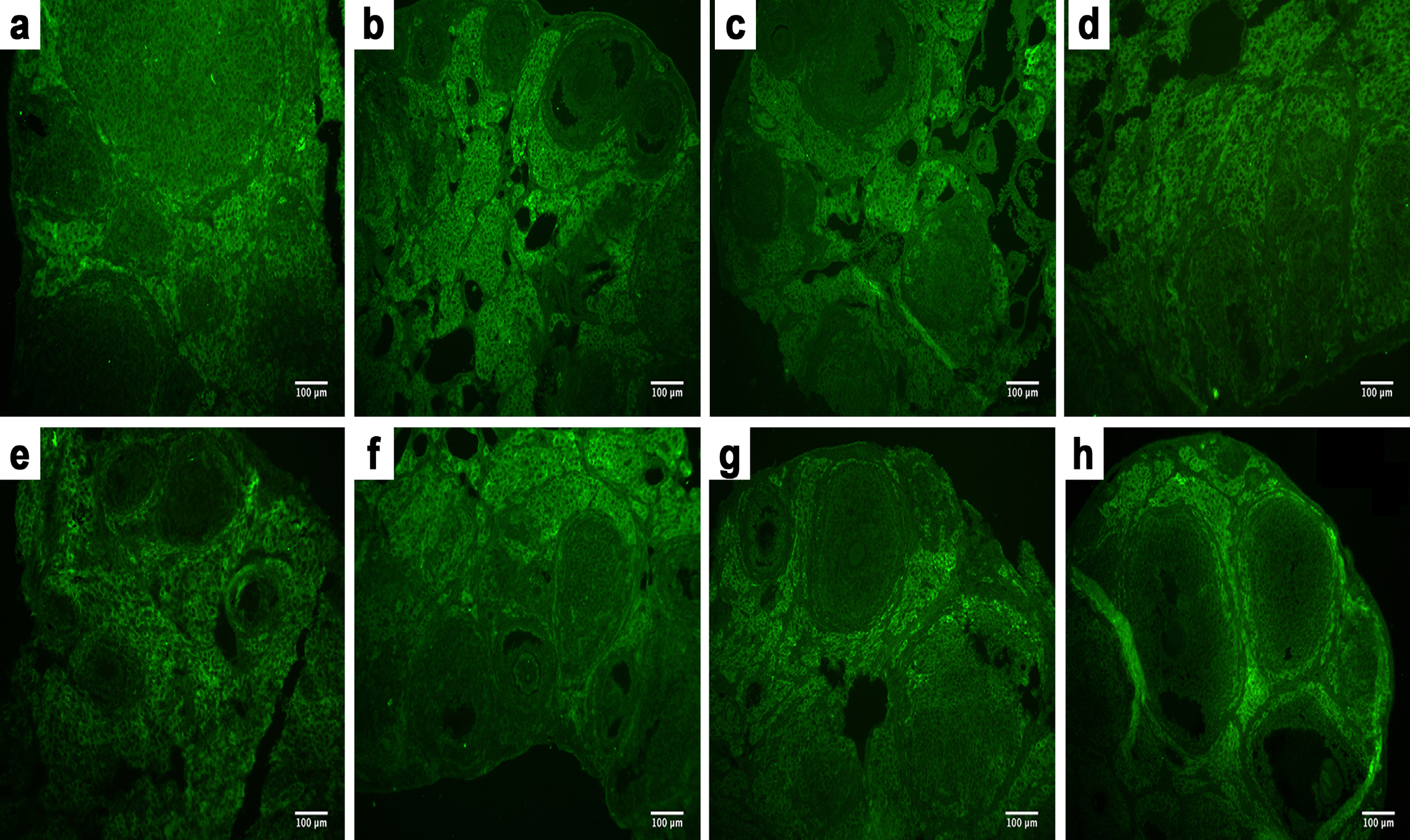

Supplement: S5 Fig — The expression and distribution of MILI (stained with FITC, green) in the control (a), group 1 (b), group 2 (c), group 3 (d), group 4 (e), group 2R (f), group 3R (g), and group 4R (h) were evaluated by immunofluorescence staining (200X magnification). Green = FITC. (TIF) [file pone.0232629.s005.tif]

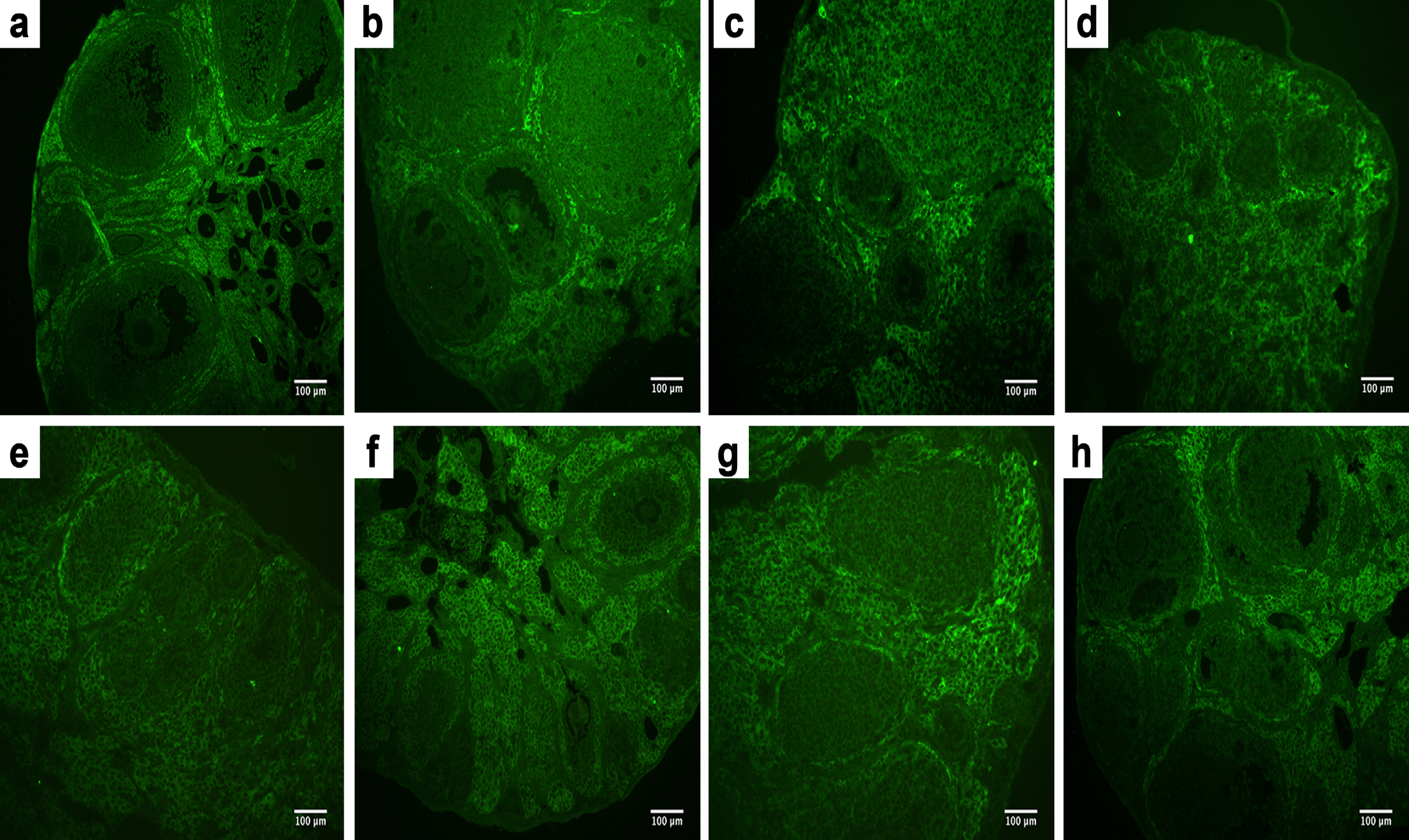

Supplement: S6 Fig — The expression and distribution of MIWI (stained with FITC, green) in the control (a), group 1 (b), group 2 (c), group 3 (d), group 4 (e), group 2R (f), group 3R (g), and group 4R (h) were evaluated by immunofluorescence staining (200X magnification).Green = FITC. (TIF) [file pone.0232629.s006.tif]
