# Supplementary material for: Effect of ovarian stimulation on the expression of piRNA pathway proteins
Source: PLoS One. 2020 May 4;15(5):e0232629. doi: 10.1371/journal.pone.0232629 (PMC7197780; doi:10.1371/journal.pone.0232629)
Supplement: Attachment — Submitted filename: Response to reviewers.doc [file pone.0232629.s008.doc]

**Response to reviewer and editor comments**

**(PONE-D-19-34298R1)**

Dear Editor,

We thank you and the reviewers for the thorough reading and constructive comments of our manuscript. We are pleased to submit the improved research article, for your consideration in the PLOS ONE. Our response to the editor and reviewer comments is as follows.

**Editor Comments**

**Comment: The manuscript is way improved, however still few errors are seen and few more points need to be explained in detail. Authors should edit the manuscript for Grammar and Plos style and also address all the comments of reviewers carefully.**

**Response**: Thank you for the comment. We carefully reviewed the manuscript again according to PLOS ONE author instructions to meet PLOS ONE’s style requirement. We have resubmitted our revised manuscript to a professional editing firm (Protranslate) for professional grammar editing. The changes are marked in red (blue in pdf file). A certificate provided from Protranslate is attached at the end of this file. A copy of our manuscript showing our changes was uploaded as a "'Revised Manuscript with Track Changes'" file, and a clean copy of the edited manuscript was uploaded as the new “manuscript" file.

**Review Comments to the Author:**

**Reviewer #1:**

**Comment 1:**
**Figure -1 - Why the Error bars are missing?**

**Response 1**: We apologize for this mistake. We have added the error bars in the Fig 2 (updated caption)*.*

**Comment 2: mili, miwi, mael, tdrd1, tdrd9 gene name/ protein names should be written in corresponding Capital or italics**

**Response 2**: Thank you for this advice. We updated the gene and protein symbols according to “MGI-Guidelines for Nomenclature of Genes, Genetic Markers, Alleles, & Mutations in Mouse & Rat” (Gene symbols generally are italicized, with only the first letter in uppercase and the remaining letters in lowercase. Protein designations are the same as the gene symbol, but are not italicized and all are upper case).

**Comment 3: Figure legends has to be detailed with subdivisions a,b, ... described and legends has to be seperate and Results and Discussion should not merely describe the numerical data. More of scientntroduction, results and discussion section.**

**Response 3**: We agree with the comment. As suggested, we revised the figure legends and citation of figures in the introduction, results, and discussion sections (Marked with pink).

**Comment 4: Less coherent information is important than more incoherent information, Hence kindly make the manuscript as simple as possible.**

**Response 4:** Thank you for the suggestion. We apologize for our confusing statement. Because some of the statements were confusing and/or redundant, we have removed (marked with green) or revised some of them (marked with yellow).

**Comment 5: S7 has to be incorporated as main figure with complete explanation of the pathway as figure legends.**

**Response 5**: We appreciate your suggestion. As per the reviewer’s advice, we have incorporated Fig S7 as the main figure (Fig 1) with a complete explanation of the pathway as figure legends and revised partially. We hope the current form will be clearer.

**Comment 6: Authors need to see other plos one papers more carefully for better formatting of the manuscript.**

**Response 6**: Thank you for the comment. We have adapted the manuscript according to PLOS ONE's requirements.

### Comment 7: Tables has to be separate not in the text.

**Response 7**: Thank you for the comment, but according to the submission guidelines, each table has to be placed in the manuscript file directly after the paragraph in which it is first cited. Tables must not be submitted in the separate files.

**Comment 8: Still needs grammar improvement - For eg In abstract line 23 - "greatest decreases " decrease**

**Response 8**: We deeply apologize for grammatical errors. Grammatical error in line 23 has been corrected. Furthermore, we have resubmitted our revised manuscript to a professional editing firm (Protranslate) for professional grammar editing. The changes are marked in red (blue in pdf file).. A certificate provided from Protranslate is attached at the end of this file.

**Reviewer #3**:

**Comment 1: The response to comment 5 was not satisfactory.**

**Response 1**: We are sorry for that comment. In order to create a more suitable figure, we have incorporated Fig S7 as the main figure (Fig 1) with a complete explanation of the pathway as figure legends and revised partially. We hope the current form will be clearer.

**Comment 2: The authors have mentioned that expression levels of Tdrd 9, Tdrd1, and Mael were less in 3R and 4R (not as significant) compared to control but 2R showed maximum suppression in expression. This data correlates with E2 levels. But the authors have not given an explanation for the observation of why gene expression is low in 2R than in 3R OR 4R. I am curious to understand if increase in repetitive rounds of OS is better than 2R. It is interesting to find decrease in E2 levels in 3R and 4R compared to 2R, what is the most logical explanation for this?**

**Response 2**: We appreciate the reviewer's comments on the subject above. We agree broadly with your consideration. Indeed, there was a significant decrease in the expression levels of these 3 genes in the 3R group. But as mentioned, the reduction rate in 2R was the highest. This highest reduction rate observed in the expression of Tdrd 9, Tdrd1 and Mael in the 2R group correlated with E2 levels suggests that there may be a complex regulation mechanism between the genes we examined and the other genes involved in piRNA biogenesis or other factors that have the potential to affect the E2 level in the repeated OS process. At first glance, increasing the number of OS cycles appears to have a positive effect on at least these 3 genes. However, to reveal the main effects of OS on this pathway in detail, we think that in different procedures further studies are needed to examine piRNA sequencing, FSH, LH, E2 hormone and receptor levels, and expression and the function of the proteins involved in the piRNA pathway. Furthermore, as we mentioned in the discussion section of our manuscript, all these findings suggest that the effect of plasma E2 levels on the expression of these proteins may vary from protein to protein in the piRNA pathway or there may be a complex regulatory pattern between these proteins.

**Comment 3: L 258: new abbreviation 'FR' should be mentioned first in L 256**

**Response 3:** We apologize for the mistake in line no. 256. We have mentioned abbreviation 'FR' (Fold regulation).
