# Supplementary material for: Effect of ovarian stimulation on the expression of piRNA pathway proteins
Source: PLoS One. 2020 May 4;15(5):e0232629. doi: 10.1371/journal.pone.0232629 (PMC7197780; doi:10.1371/journal.pone.0232629)
Supplement: Attachment — Submitted filename: Response to reviewers.doc [file pone.0232629.s009.doc]

**Response to reviewer and editor comments**

**(PONE-D-19-34298R2)**

Dear Editor,

First of all, we would like to thank the editor and all of the reviewers for their careful review of the manuscript and professional comments and suggestions for improving our initial manuscript. We are pleased to submit the improved research article, for your consideration in the PLOS ONE. Our response to the reviewer's comments is as follows.

**Reviewer #1:**

**Comment 1-Line number 117- decreasing symbols has to be removed. Punctuation has to be checked.**

**Response 1**-Thank you for this advice. As your advice, line number 117- decreasing symbols has been removed from the manuscript. Punctuation has been checked and some corrections made in the manuscript (marked with red).

**Comment 2**-**MITOPLD, MILI, TDRD..etc protein names has to be consistent through out the manuscript when it comes to deciding uppercase or lower case.**

**Response 2-** We deeply apologize for our inattention that caused inconsistency about this issue. We carefully reviewed the manuscript again according to MGI-guidelines for the nomenclature of genes and proteins. We made some corrections in the manuscript, and figure 2 according to the MGI-guidelines.

**Comment 3**-**Figure -1 needs figure legends.**

**Response 3-** Thanks for your suggestion. As suggested, Fig.1 legends have added to the manuscript.

**Comment** 4-In Figure-1 typo “ Secondery”

We apologize for this misspelling. We have corrected this word in the Fig.1

**Reviewer #3: :**

**Comment 1**-**Few suggestions: check the use of articles and punctuation in figure 1 legend. Also cite references for explaining fig1.**

**Response 1-** Thanks for your suggestion. The punctuation in Fig.1 legends and the manuscript were rechecked and corrections were made (marked with red). Furthermore, references have been added for the description of fig 1.
